# PM2.5 triggers tau aggregation in a mouse model of tauopathy

**DOI:** 10.1172/jci.insight.176703

**Published:** 2024-07-22

**Authors:** Congcong Liu, Lanxia Meng, Yan Gao, Jiehui Chen, Min Zhu, Min Xiong, Tingting Xiao, Xiaoling Gu, Chaoyang Liu, Tao Li, Zhentao Zhang

**Affiliations:** 1 Department of Neurology, Renmin Hospital of Wuhan University, Wuhan, China.; 2 Research Center for Environment and Health, Zhongnan University of Economics and Law, Wuhan, China.; 3 TaiKang Center for Life and Medical Sciences, Wuhan University, Wuhan, China.; 4 Shandong First Medical University (Shandong Academy of Medical Sciences), Jinan, China.

**Keywords:** Neuroscience, Alzheimer disease

## Abstract

The aggregation and prion-like propagation of tau are the hallmarks of Alzheimer’s disease (AD) and other tauopathies. However, the molecular mechanisms underlying the assembly and spread of tau pathology remain elusive. Epidemiological data show that exposure to fine particulate matter (PM2.5) is associated with an increased risk of AD. However, the molecular mechanisms remain unknown. Here, we showed that PM2.5 triggered the aggregation of tau and promoted the formation of tau fibrils. Injection of PM2.5-induced tau preformed fibrils (PFFs) into the hippocampus of tau P301S transgenic mice promoted the aggregation of tau and induced cognitive deficits and synaptic dysfunction. Furthermore, intranasal administration of PM2.5 exacerbated tau pathology and induced cognitive impairment in tau P301S mice. In conclusion, our results indicated that PM2.5 exposure promoted tau pathology and induced cognitive impairments. These results provide mechanistic insight into how PM2.5 increases the risk of AD.

## Abstract 2

PM2.5 induces tau preformed fibrils that exacerbate Alzheimer’s disease–like phenotypes in mouse models.

## Introduction

Tauopathies are a group of neurodegenerative disorders that are characterized by tau deposits in the brain. Alzheimer’s disease (AD) is the most common tauopathy, affecting more than 30 million people worldwide (1). Tau is a microtubule-associated protein that is abundantly expressed in neurons. It regulates microtubule polymerization, neuronal polarity, axonal outgrowth, and axonal transport (2–4). Physiological tau is a natively unfolded protein that lacks a secondary structure. In the brains of patients with tauopathies, tau is hyperphosphorylated and aggregated into β-sheet structures that deposit in neurons (5, 6). Converging lines of evidence support that tau pathology spreads in the brain through prion-like propagation, in which tau fibrils act as a template to recruit soluble tau for further tau aggregation (7–9). The extent of tau pathology correlates with cognitive dysfunction in patients with AD (10). Interestingly, tau fibrils isolated from different patients have unique conformations and pathologic activities, known as tau stains (11–13). However, it remains largely unknown what regulates the aggregation and spreading of tau during the onset of AD.

Air pollution, a major environmental risk to human health, is defined as a complicated mixture of various particulate matter (PM), lead, and gases (sulfur dioxide, nitrogen dioxide, carbon monoxide, and so on). Fine particulate matter (PM2.5), particles with diameters less than 2.5 μm, is now recognized as a risk factor for AD (14). It has been reported that PM2.5 can cross the blood-brain barrier and accumulate in the brain (15). PM2.5 exposure triggers neuronal neuroinflammation, induces cognitive dysfunction, and is associated with AD-related neuropathology and cognitive outcomes (16, 17). A longitudinal cohort study from the United States found that PM2.5 exposure increases the incidence of AD (14). Young Mexico City metropolitan residents exposed to high levels of PM2.5 showed frontal tau hyperphosphorylation (18). However, it remains unknown whether PM2.5 directly induces tau aggregation and neurodegeneration.

In this report, we show that PM2.5 promotes tau aggregation both in vitro and in vivo. Moreover, the PM2.5-seeded tau fibrils showed enhanced seeding activity and neurotoxicity compared with pure tau fibrils. Inhalation of PM2.5 accelerates tau pathology in tau P301S transgenic mice. Taken together, our results indicate that PM2.5 may be a trigger of tau pathology.

## Results

### PM2.5 accelerates tau aggregation.

**Figure 1:**
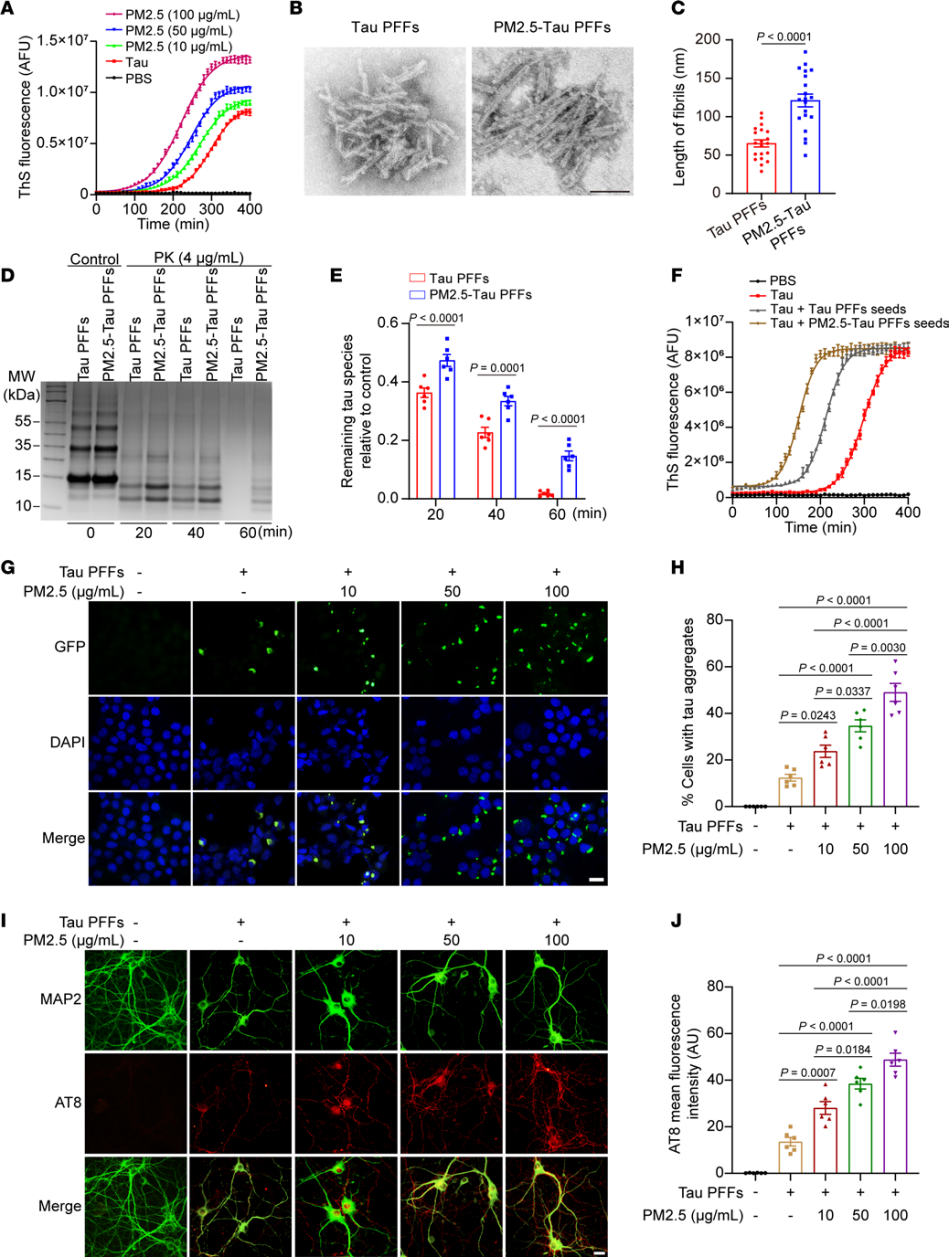
Fine particulate matter (PM2.5) promotes tau aggregation in vitro. (**A**) The kinetics of tau aggregation in the presence of different concentrations of PM2.5. AFU, arbitrary fluorescence units. *n* = 7 biologically independent experiments. (**B** and **C**) The ultrastructure of tau preformed fibrils (PFFs) and PM2.5-tau PFFs. (**B**) Transmission electron microscopy (TEM) analysis. Scale bar: 100 nm. (**C**) Quantitation of the lengths of the fibrils. *n* = 20 randomly selected images from 6 independent biological experiments. (**D** and **E**) PK digestion of tau PFFs and PM2.5-tau PFFs. (**D**) Equal amounts of tau PFFs and PM2.5-tau PFFs were incubated with 4 μg/mL PK, then analyzed by Coomassie blue staining. (**E**) The bar graph shows the quantification of the remaining tau species relative to control. *n* = 6 biologically independent experiments. (**F**) Tau aggregation kinetics induced by 5% tau PFFs and PM2.5-tau PFFs. *n* = 7 biologically independent experiments. (**G** and **H**) Tau-HEK293 cells were pretreated with different concentrations of PM2.5 for 24 hours, then transduced with tau PFFs. (**G**) Shown are insoluble tau aggregates at 48 hours after transduction. Scale bar: 20 μm. (**H**) Quantification of insoluble tau aggregates. *n* = 6 independent biological experiments (each point represents the average of 10 random fields from each experiment). (**I** and **J**) Primary neurons were pretreated with PM2.5 for 24 hours, then transduced with tau PFFs. (**I**) Shown are p-tau (AT8) staining at 10 days after transduction. Scale bar: 20 μm. AU, arbitrary units. (**J**) Quantification of AT8 fluorescence intensity. *n* = 6 independent biological experiments (each point represents the average of 10 random fields from each experiment). Data are presented as mean ± SEM. *P* values were determined by Student’s *t* test (**C**), 2-way ANOVA followed by Holm-Šídák multiple comparisons test (**E**), or 1-way ANOVA followed by Tukey’s multiple comparisons test (**H** and **J**).

To explore the effect of PM2.5 on the aggregation of tau, we incubated monomeric tau repeat domain (RD) (K18, 1 mg/mL) in the presence or absence of PM2.5 and monitored the aggregation kinetics using thioflavin S (ThS) assay. The results showed that PM2.5 accelerated the aggregation of K18 in a concentration-dependent manner (Figure 1A). To investigate whether tau preformed fibrils (PFFs) interact with PM2.5, we centrifuged the mixture at 500 rpm to spin down PM2.5. Interestingly, most of the tau species remained in the supernatant in the absence of PM2.5, while tau was found in the pellet fraction with PM2.5 (Supplemental Figure 1, A and B;; supplemental material available online with this article; https://doi.org/10.1172/jci.insight.176703DS1). To explore whether PM2.5 changes the properties of pure tau PFFs and tau PFFs formed in the presence of PM2.5 (PM2.5-tau PFFs), we observed the morphology of pure tau PFFs and PM2.5-tau PFFs using transmission electron microscopy. The results showed that PM2.5-tau PFFs were longer than pure tau PFFs (Figure 1, B and C). We further tested the degradation of pure tau PFFs and PM2.5-tau PFFs using proteinase K (PK) digestion assay. Interestingly, PM2.5-tau PFFs were more resistant to PK digestion than pure tau PFFs (Figure 1, D and E). To investigate whether PM2.5 affects the seeding activity of tau PFFs, we added equal amounts of PM2.5-tau PFFs and pure tau PFFs into tau monomers and monitored the aggregation of tau. ThS assay showed that PM2.5-tau PFFs were more potent at seeding the aggregation of monomeric tau than pure tau PFFs (Figure 1F), indicating that PM2.5-tau PFFs have enhanced seeding activity. We further tested the effect of PM2.5 on the aggregation of tau in HEK293 cells stably expressing GFP-tagged tau RD (tau-HEK293 cells). As reported before (8), transduction of tau PPFs induced the formation of insoluble aggregates in tau-HEK293 cells. The cells were pretreated with different concentrations of PM2.5 for 24 hours and then transduced with tau PFFs. The results showed that pretreatment with PM2.5 increased the formation of aggregates in a concentration-dependent manner (Figure 1, G and H). However, the filter paper extracts did not promote tau aggregation (Supplemental Figure 2, A and B). Similarly, transduction of tau PFFs induced the phosphorylation of tau in primary cultured neurons. The levels of phosphorylated tau (p-tau) increased in neurons pretreated with PM2.5 (Figure 1, I and J). These results indicate that PM2.5 promotes the aggregation of endogenous tau.

### PM2.5 enhances the seeding activity and neurotoxicity of tau PFFs.

**Figure 2:**
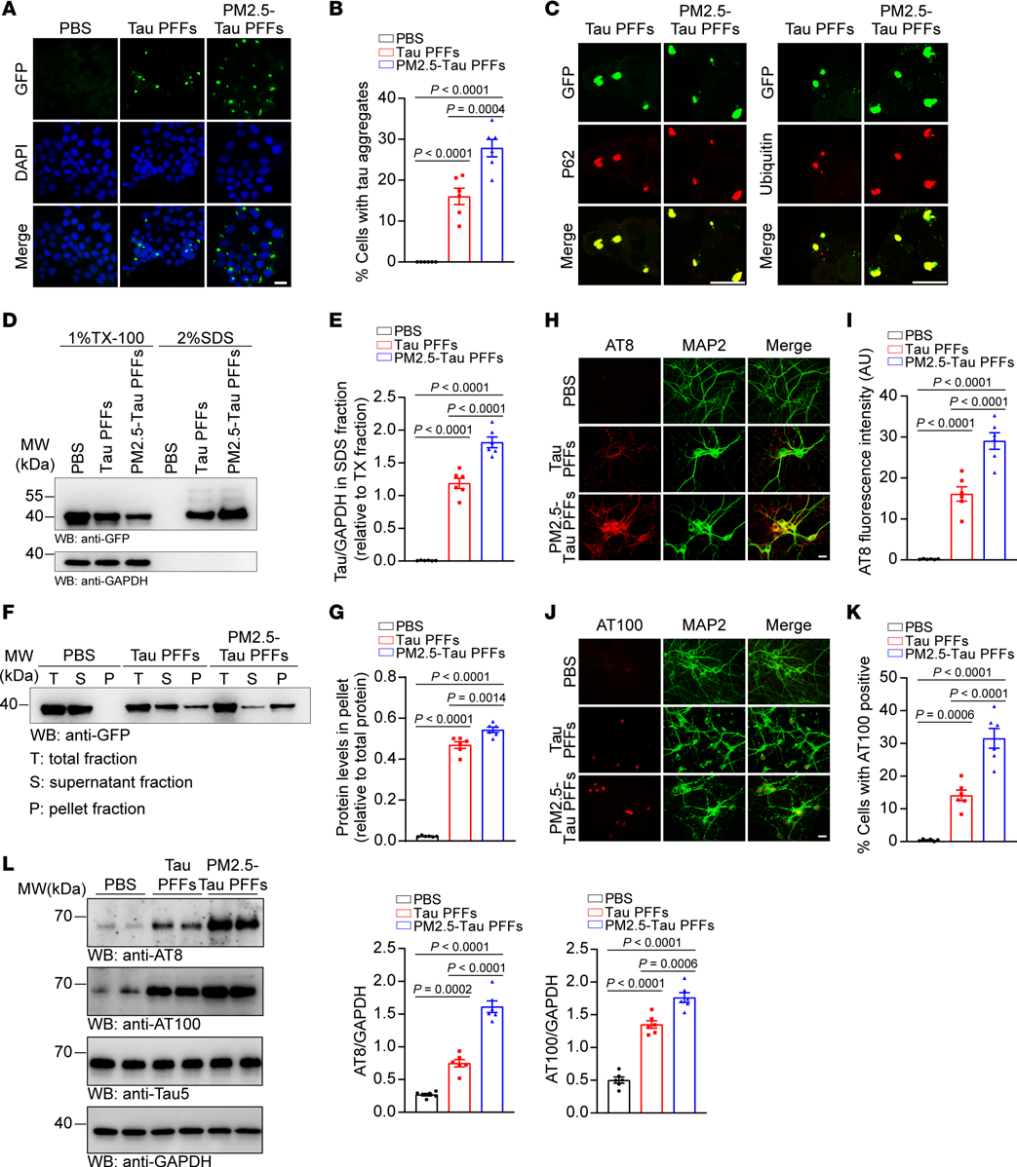
PM2.5-tau PFFs show enhanced seeding activity in vitro. (**A** and **B**) Tau aggregates in tau-HEK293 cells with different treatments. (**A**) Shown are insoluble tau aggregates at 48 hours of transduction. (**B**) Quantification of tau aggregates. *n* = 6 independent biological experiments (each point represents the average of 10 random fields from each experiment). (**C**) Immunofluorescence showing that tau aggregates colocalize with ubiquitin and p62 in tau-HEK293 cells. (**D** and **E**) Tau-HEK293 cells with different treatments were sequentially extracted with 1% Triton X-100 in TBS with protease and phosphatase inhibitors (TX-100) lysis buffer and 2% SDS lysis buffer. The bar graph shows the quantitative analysis of tau level. *n* = 6 biologically independent experiments. (**F** and **G**) Sedimentation analysis of tau-HEK293 cells transduced with different treatments. The bar graph shows the quantification of tau levels in the pellets relative to total tau. *n* = 6 biologically independent experiments. (**H** and **I**) Representative images of p-tau (AT8) immunostaining in primary neurons with different treatments for 10 days. (**H**) p-tau (AT8) staining. (**I**) The bar graph shows the quantification of AT8. *n* = 6 independent biological experiments (each point represents the average of 10 random fields from each experiment). (**J** and **K**) Representative images of p-tau (AT100) in primary neurons with different treatments for 10 days. (**J**) p-tau (AT100) staining. (**K**) The bar graph shows the quantification of AT100. *n* = 6 independent biological experiments (each point represents the average of 10 random fields from each experiment). (**L**) Western blot assay of AT8, AT100, and total tau levels in neurons treated with different treatments. The bar graphs are the quantification of AT8 and AT100 levels relative to GAPDH. *n* = 6 biologically independent experiments. Data are presented as mean ± SEM. *P* values were determined by 1-way ANOVA followed by Tukey’s multiple comparisons test. Scale bar: 20 μm.

We further compared the seeding activity of tau PFFs and PM2.5-tau PFFs in tau-HEK293 cells. When equal amounts of tau PFFs and PM2.5-induced tau PFFs were transduced into tau-HEK293 cells, more aggregates were formed in cells transduced with PM2.5-tau PFFs (Figure 2, A and B). Furthermore, the aggregates induced by tau PFFs or PM2.5-tau PFFs were positive for p62 and ubiquitin (Figure 2C). Sequential extraction experiments showed that more high–molecular weight tau species that were not soluble in 1% Triton X-100 in TBS with protease and phosphatase inhibitors (TX-100) were found in cells transduced with PM2.5-tau PFFs than in cells transduced with pure tau PFFs (Figure 2, D and E). Likewise, sedimentation analysis found more tau species in the pellet fraction of cells transduced with PM2.5-tau PFFs than in cells transduced with tau PFFs (Figure 2, F and G).

We further compared the effect of pure tau PFFs and PM2.5-tau PFFs on tau phosphorylation in primary neurons. When equal amounts of pure tau PFFs and PM2.5-tau PFFs were added to the primary neurons, PM2.5-tau PFFs induced higher levels of tau phosphorylation (stained with AT8 and AT100 antibodies) than tau PFFs (Figure 2, H–K). These results were verified by Western blot analysis (Figure 2L). Synaptic degeneration occurs at the early stage of AD and contributes to cognitive impairment (19). Tau fibrils have been reported to cause microtubule depolymerization (20–22) and be toxic to synapses and neurons (23–25). Immunostaining with anti-MAP2 showed that the morphology of microtubules was altered after the neurons were exposed to PM2.5-tau PFFs (Figure 1I and Figure 2, H and J). Thus, we further investigated the effect of PM2.5-tau PFFs on synapses in primary neurons. Western blot analysis showed that PM2.5-tau PFFs induced a greater decrease in synaptic proteins, including synapsin I, synaptophysin, and PSD95, than pure tau PFFs (Supplemental Figure 3A). DiI staining was performed to evaluate the density of dendritic spines in neurons. The density of dendritic spines was decreased in neurons transduced with PM2.5-tau PFFs compared with neurons transduced with tau PFFs (Supplemental Figure 3B). As expected, tau PFFs induced apoptosis of neurons, and this effect was more pronounced with PM2.5-tau PFFs as indicated by TUNEL staining (Supplemental Figure 3C). These results indicate that PM2.5-induced tau fibrils are more potent at seeding endogenous tau and inducing neurotoxicity in neurons.

### PM2.5-tau PFFs enhance tau pathology and neuroinflammation in vivo.

**Figure 3:**
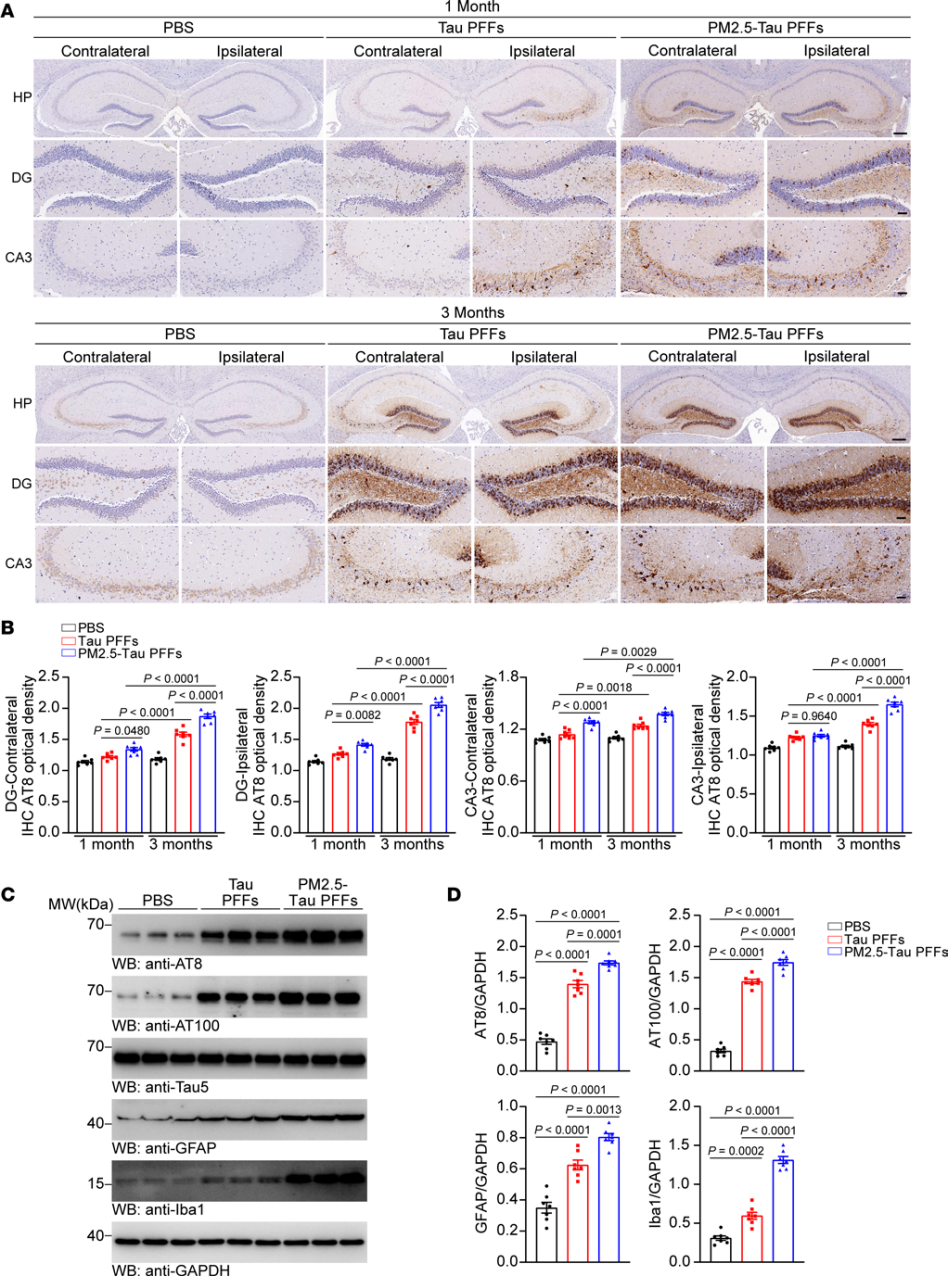
PM2.5-tau PFFs promote the propagation of tau pathology in vivo. (**A** and **B**) Immunohistochemistry of p-tau (AT8) in tau P301S mice after the injection of PBS, tau PFFs, or PM2.5-tau PFFs. (**A**) Representative images of p-tau (AT8) staining in the dentate gyrus (DG) and Cornu Ammonis 3 (CA3) of the mouse hippocampus at 1 month and 3 months (scale bars, 100 μm in the top panel, 20 μm in the lower panels). (**B**) Bar graphs are the quantification of average optical densities of AT8. *n* = 7 mice per group. (**C**) Representative immunoblots of p-tau (AT8 and AT100), total-tau, GFAP, and Iba1 in the hippocampus of mice injected with PBS, tau PFFs, or PM2.5-tau PFFs at 3 mpi. (**D**) The bar graphs show the quantification of AT8, AT100, GFAP, and Iba1 relative to GAPDH. *n* = 7 mice per group. Data are presented as mean ± SEM. *P* values were determined by 1-way ANOVA followed by Tukey’s multiple comparisons test.

To investigate whether PM2.5 enhances the seeding activity of tau PFFs in vivo, we injected 5 μg of tau PFFs or PM2.5-tau PFFs into the unilateral hippocampus of tau P301S transgenic mice. Tau pathology was assessed at 1 and 3 months postinjection (mpi). Immunostaining with AT8 and AT100 antibodies showed that tau pathology increased in a time-dependent manner in mice injected with tau PFFs and PM2.5-tau PFFs. In agreement with what has been observed in primary neurons, PM2.5-tau PFFs induced more abundant tau pathology in the hippocampus than pure tau PFFs (Figure 3, A and B). However, it did not trigger tau phosphorylation in WT mice (Supplemental Figure 4), which was consistent with previous reports (26–30). Western blot analysis found that the levels of p-tau were higher in the brain lysates from the PM2.5-tau PFF–injected mice than in those from tau PFF–injected mice (Figure 3, C and D). Neuroinflammation contributes to neurodegeneration in AD. Thus, we further examined the effect of PM2.5-tau PFFs on the activation of microglia and astrocytes. We found that the levels of the microglia marker ionized calcium-binding adapter molecule 1 (Iba1) and astrocyte marker glial fibrillary acidic protein (GFAP) in the hippocampal lysates of PM2.5-tau PFF–injected mice were higher than those in tau PFF–injected mice at 3 mpi (Figure 3, C and D). Together, these results indicate that PM2.5-tau PFFs trigger more severe tau pathology and neuroinflammation than tau PFFs in vivo.

### PM2.5-tau PFFs induce memory deficits and synaptic dysfunction in tau P301S mice.

**Figure 4:**
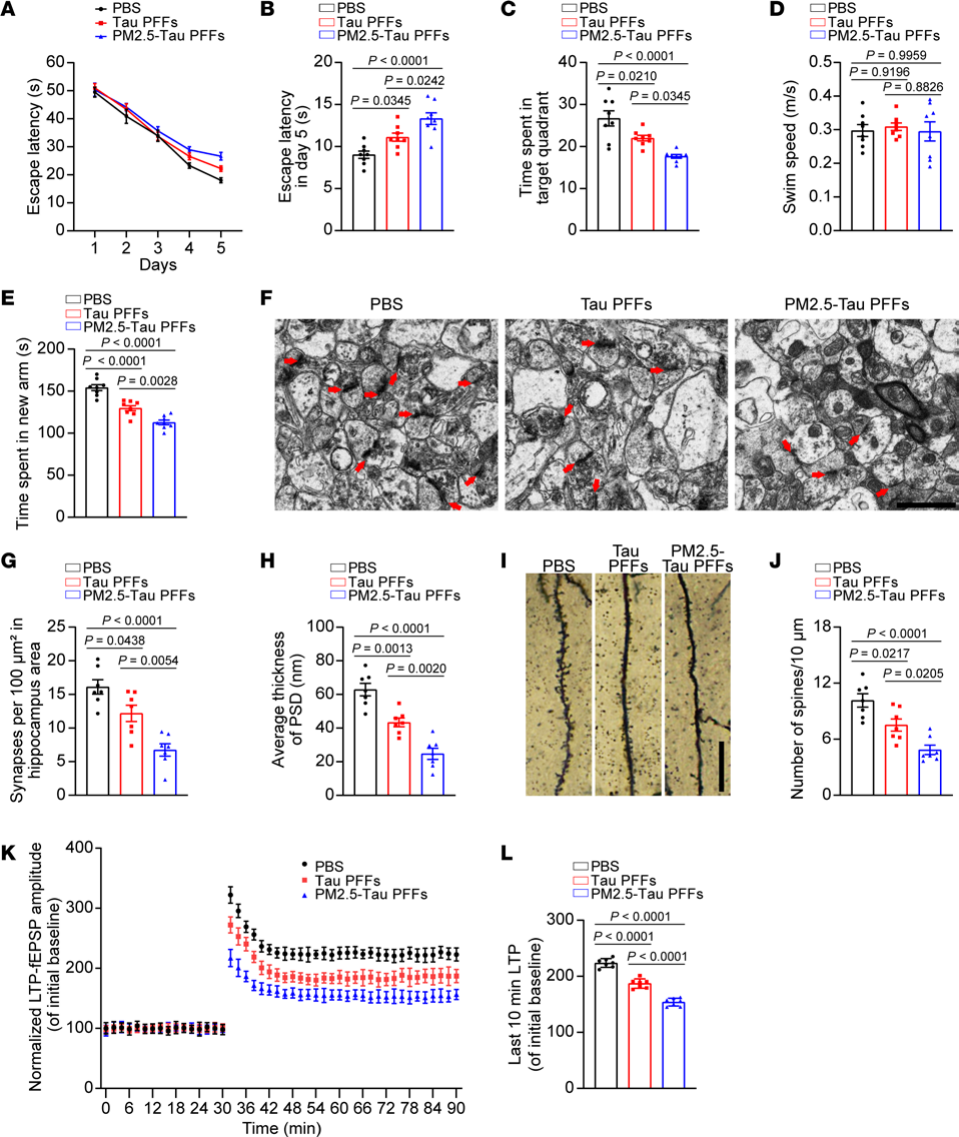
PM2.5-tau PFFs induce cognitive impairment and synaptic dysfunction in tau P301S mice. (**A**–**D**) The spatial learning memory of mice injected with PBS, tau PFFs, or PM2.5-tau PFFs was assessed by the Morris water maze test. Shown are the escape latency during training (**A**), the escape latency on day 5 (**B**), the time spent in the target quadrant in the probe trial (**C**), and the swim speed (**D**) of mice injected with PBS, pure tau PFFs, or PM2.5-tau PFFs. *n* = 8 mice per group. (**E**) The spatial working memory of mice injected with PBS, tau PFFs, or PM2.5-tau PFFs was assessed by the Y-maze test. Shown is the time spent in the new arm. *n* = 8 mice per group. (**F**–**H**) Representative images of synapses in the hippocampus of mice injected with PBS, pure tau PFFs, or PM2.5-tau PFFs (scale bars, 1 μm). Quantification of synapse clefts (**G**) and postsynaptic density (PSD) (**H**). *n* = 7 mice per group (each point represents the average of 10 random fields from each mouse). (**I** and **J**) Representative images of Golgi staining of the dendritic spines in the hippocampal area. (**J**) Quantification of spine density. *n* = 7 mice per group (each point represents the average of 10 random fields from each mouse). Scale bars, 20 μm. (**K** and **L**) The amplitude of fEPSPs after HFS recorded in hippocampal slices. (**K**) Shown traces are representative fEPSPs of 7 samples recorded before and after LTP induction. (**L**) Quantitative analysis of normalized fEPSPs 50–60 minutes after HFS. *n* = 7 mice per group. Data are presented as mean ± SEM. *P* values were determined by 1-way ANOVA followed by Tukey’s multiple comparisons test.

To illustrate the effect of PM2.5-tau PFFs on the memory of tau P301S mice, we performed the Morris water maze test. During the training phase, the latency to find the platform gradually decreased in all mice, showing a learning effect. Compared with mice injected with pure tau PFFs, mice injected with PM2.5-tau PFFs showed more severe learning impairment (Figure 4, A and B). In the probe trial, mice injected with PM2.5-tau PFFs spent less time in the target quadrant than mice injected with pure tau PFFs, suggesting that PM2.5-tau PFFs induce more severe memory deficits (Figure 4C). The mice with different treatments showed comparable swim speeds, indicating that different treatments did not affect motor function (Figure 4D). Similarly, in the Y-maze test, mice injected with PM2.5-tau PFFs also spent less time in the new arm (Figure 4E), indicating that PM2.5-tau PFFs induce more severe deficits in spatial memory.

Synaptic degeneration occurs early during the progression of AD and is considered the basis of cognitive impairment (31). We further determined the effect of PM2.5-tau PFFs on synapses using electron microscopy. The density of synapses decreased in mice injected with tau PFFs, indicating synaptic degeneration. The loss of synapses was more severe in the hippocampus of mice injected with PM2.5-tau PFFs (Figure 4, F and G). Moreover, the thickness of the postsynaptic membrane was decreased in mice injected with PM2.5-tau PFFs (Figure 4H). In addition, Golgi staining showed that PM2.5-tau PFFs induced more loss of dendritic spines (Figure 4, I and J). These results indicate that PM2.5-tau PFFs induce more severe synaptic degeneration.

The long-term potentiation (LTP) of field excitatory postsynaptic potentials (fEPSPs) in the hippocampus is believed to be the basis of learning and memory (32). We further investigated the LTP of fEPSPs in the hippocampal region, which represents synaptic plasticity. The results showed that LTP was diminished in mice injected with tau PFFs and PM2.5-tau PFFs, with PM2.5-tau PFFs showing more severe detrimental effect on LTP (Figure 4, K and L). In summary, these results indicate that PM2.5-tau PFFs induce more severe cognitive impairments and synaptic dysfunction.

### Intranasal delivery of PM2.5 induces tau pathology and memory deficits in tau P301S mice.

**Figure 5:**
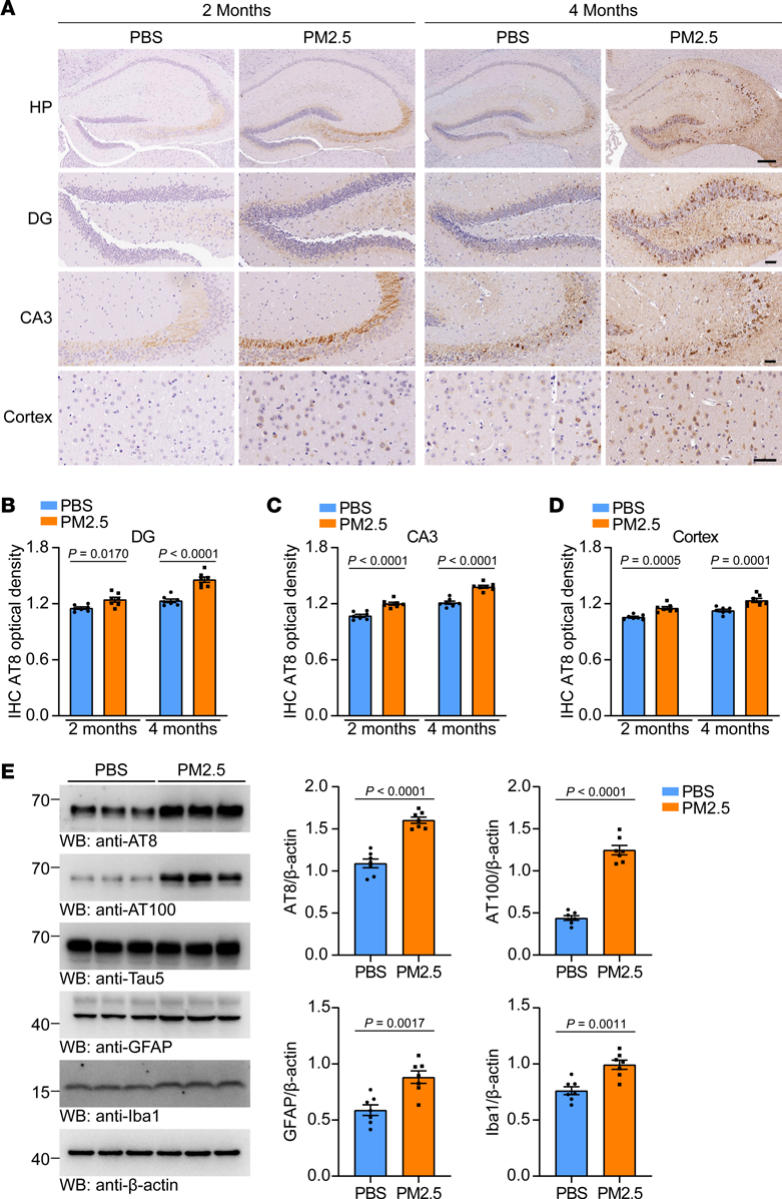
Intranasal instillation of PM2.5 triggers tau pathology in tau P301S mice. (**A**–**D**) Immunohistochemistry of p-tau (AT8) in tau P301S mice treated with PM2.5 for 2 months and 4 months. (**A**) Representative images of p-tau pathology (AT8) in the DG, CA3, and cortex of mice (scale bar, 100 μm for images of HP, 50 μm for images of the DG, CA3, and cortex). Quantification of p-tau pathology in the DG (**B**), CA3 (**C**), and cortex (**D**). *n* = 7 mice per group. (**E**) Representative immunoblots of p-tau (AT8 and AT100), total tau, GFAP, and Iba1 in the hippocampus of mice treated with PBS or PM2.5 for 4 months. The bar graphs show the quantification of AT8, AT100, GFAP, and Iba1 relative to GAPDH. *n* = 7 mice per group. Data are presented as mean ± SEM. *P* values were determined by 2-way ANOVA followed by Holm-Šídák multiple comparisons test (**B**–**D**) or Student’s *t* test (**E**).

The nasal cavity is the main route for PM2.5 to invade the human body (33). To determine whether PM2.5 contributes to tau pathology in vivo, we further tested whether intranasal administration of PM2.5 induces AD-like phenotypes. Two-month-old tau P301S mice received intranasal instillation of PM2.5 or PBS. Two months later, immunostaining showed obvious p-tau signals in the hippocampus of mice treated with PM2.5. However, few p-tau signals were detected in mice that received PBS. Four months after PM2.5 administration, tau pathology was more abundant in the hippocampus of mice treated with PM2.5 (Figure 5, A–D). Furthermore, inhalation of PM2.5 did not induce tau pathology in WT mice (Supplemental Figure 5), which is consistent with previous reports (34, 35). Western blotting also verified that PM2.5 accelerated tau phosphorylation in tau P301S mice (Figure 5E). Furthermore, the levels of Iba1 and GFAP in the hippocampal region were higher in mice that inhaled PM2.5 than in control mice (Figure 5E).

**Figure 6:**
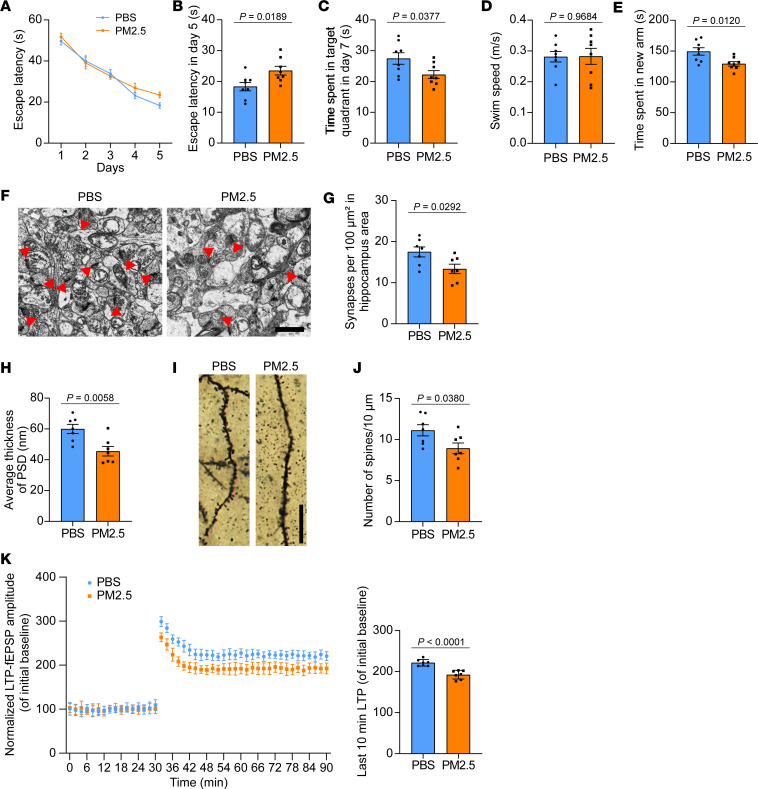
PM2.5 induces cognitive impairment and synaptic dysfunction in tau P301S mice. (**A**–**C**) The spatial learning memory of mice that received intranasal instillation of PBS or PM2.5 was assessed by the Morris water maze test. Shown are the escape latency during training (**A**), the escape latency on day 5 (**B**), the time spent in the target quadrant in the probe trial (**C**), and the swim speed of mice (**D**). *n* = 8 mice per group. (**E**) The spatial working memory of mice that received intranasal instillation of PBS or PM2.5 was assessed by the Y-maze test. Shown is the time spent in the new arm. *n* = 8 mice per group. (**F**–**H**) Representative images of synapses in the hippocampus of mice that received intranasal instillation of PBS and PM2.5 (**F**). Quantification of synapse clefts (**G**) and PSD (**H**). *n* = 7 mice per group (each point represents the average of 10 random fields from each mouse). Scale bars, 1 μm. (**I** and **J**) Representative images of Golgi staining of the dendritic spines in the hippocampal area (**I**). The bar graph shows the quantification of spine density (**J**). *n* = 7 mice per group (each point represents the average of 10 random fields from each mouse). Scale bars, 20 μm. (**K**) The amplitude of fEPSPs after high-frequency stimulation (HFS) recorded in hippocampal slices of mice that received intranasal instillation of PBS and PM2.5. Shown traces are representative fEPSPs of 7 samples recorded before and after LTP induction (**K**). The bar graph shows the quantitative analysis of normalized fEPSPs 50–60 minutes after HFS. *n* = 7 mice per group. Data are presented as mean ± SEM. *P* values were determined by Student’s *t* test.

We further investigated the effect of PM2.5 on the memory of tau P301S mice using the Morris water maze test. During training, the escape latency was longer in mice treated with PM2.5 than in mice treated with PBS. In the probe trial, PM2.5-treated mice also spent less time in the probe test, indicating impaired learning and memory (Figure 6, A–D). Moreover, mice that inhaled PM2.5 spent less time in the new arm in the Y-maze test, indicating that PM2.5 induces deficits in spatial memory in tau P301S mice (Figure 6E). Electron microscopy revealed that the density of synapses and thickness of PSD were decreased in mice treated with PM2.5 (Figure 6, F–H). Golgi staining revealed that the density of dendritic spines in mice treated with PM2.5 was lower than that in control mice (Figure 6, I and J). Consistently, LTP analysis verified that PM2.5 reduced synaptic plasticity in tau P301S (Figure 6K). Taken together, intranasal instillation of PM2.5 induces tau pathology, inflammatory response, synaptic dysfunction, and memory deficits in tau P301S mice.

## Discussion

Although increasing evidence indicates that PM2.5 is a risk factor for AD, whether and how PM2.5 affects tau pathology remain unclear. In the present study, we identified that PM2.5 interacts with tau and promotes its aggregation. PM2.5-seeded tau fibrils show unique morphologies and pathological properties, which are more potent at inducing tau pathology than pure tau fibrils in vitro and in vivo, causing enhanced seeding activity and neurotoxicity. Furthermore, inhalation of PM2.5 accelerates tau pathology in tau P301S mice. Our data provide evidence that PM2.5 may act as a trigger for human tau aggregation and contribute to the onset of AD.

Epidemiologic studies have shown that PM2.5 exposure increases the risk of AD (36, 37). A longitudinal cohort study showed that the first hospital admission for AD was positively correlated with the concentration of ambient PM2.5, which suggests a concentration-dependent relationship between exposure to PM2.5 and the incidence of AD (14). Moreover, Lilian et al. reported that young Mexico City metropolitan area residents exposed to PM2.5 exhibit tau hyperphosphorylation in the frontal lobe (18). However, the mechanisms underlying PM2.5-induced tau pathology remain unclear. Most studies suggest that PM2.5 can destroy the integrity of the blood-brain barrier (38) and induce neuroinflammation (39), neuronal degeneration, and cognitive dysfunction (40). To the best of our knowledge, there is no report on whether PM2.5 directly regulates tau aggregation. Here we provide evidence that PM2.5 has a direct impact on tau aggregation.

Tau fibrils extracted from different tauopathies show distinct conformational and biological properties (6, 41–44), indicating that different tau fibril structures may determine its seeding activity and neurotoxicity, which contribute to the heterogeneity of tauopathies (45, 46). Both the original seeds and cellular environments may contribute to the formation of different tau strains. Thus, different triggers may induce distinct tau strains and lead to the pathological and clinical heterogeneity of tauopathies. In this study, we observed that PM2.5-induced tau PFFs exhibited distinct ultrastructure morphologies compared with pure tau PFFs and showed the enhanced seeding activity and neurotoxicity.

Under natural conditions, the nasal mucosa is one of the most important routes by which PM2.5 invades the brain. Kang et al. reported that PM2.5 can cross the blood-brain barrier and deposit in the brain (39). Hyperphosphorylated tau was identified in the olfactory bulb of toddlers living in highly polluted metropolitan Mexico City (47). In our experiment, intranasal inhalation of PM2.5 to tau P301S mice accelerated tau pathology, synaptic degeneration, and cognitive dysfunction. These observations are consistent with the epidemiological and pathological observations. It is worth mentioning that everyone is exposed to PM2.5 over the course of life, but not all will develop AD. The protein quality control system helps exclude protein fibrils. The imbalance between the production and clearance of protein aggregates may control the transition from normal aging to tau pathology. The severity and persistence of PM2.5 exposure may determine the outcome.

In conclusion, our results support that PM2.5 may be involved in the pathogenesis of AD by directly interacting with tau and promoting its aggregation, causing synaptic dysfunction and cognitive impairment. Therefore, reducing the emission and inhalation of pollutants may be an effective measure to retard the development and progression of AD.

## Methods

### Sex as a biological variable.

Our study examined male and female animals, and similar findings were reported for both sexes.

### Antibodies and reagents.

The following antibodies were used in this study: AT8 (Thermo Fisher Scientific, MN1020), AT100 (Thermo Fisher Scientific, MN1060), His (Proteintech, 66005-1-Ig), MAP2 (Proteintech, 17490-1-AP, and Invitrogen, 13-1500), P62 (Abcam, 56416), Ubiquitin (Cell Signaling Technology, 3936S), GFP (Proteintech, 66002-1-Ig), GAPDH (Proteintech, 60004-1-Ig), β-actin (Proteintech, 66009-1-Ig), tau (Thermo Fisher Scientific, MA5-12808), Iba1 (Wako, 019-19741), GFAP (Invitrogen, PA5-16291), Synapsin I (Proteintech, 20258-1-AP), Synaptophysin (Proteintech, 17785-1-AP), PSD95 (Proteintech, 20665-1-AP), Alexa Fluor 488–Goat anti-Mouse (Invitrogen, A11001), Alexa Fluor 488–Goat anti-Rabbit (Invitrogen, A11034), Alexa Fluor 594–Goat anti-Mouse (Invitrogen, A11005), Alexa Fluor 594–Goat anti-Rabbit (Invitrogen, A11012), DAPI (Biofroxx, EZ3412B205), HRP-conjugated anti-mouse IgG (Bio-Rad, 170-6516), and HRP-conjugated anti-rabbit IgG (Bio-Rad, 170-6515). The TUNEL in situ cell death detection kit was purchased from Roche (11684795910). DiI was purchased from Invitrogen (D-282).

### Mice.

Specific pathogen–free (SPF) tau P301S transgenic mice (line PS19) and WT C57BL/6J mice were obtained from The Jackson Laboratory (stock numbers: 008169 and 000664, respectively). The mice were kept under SPF conditions at a 12-hour light/12-hour dark cycle at 22°C and fed food and water ad libitum. Two-month-old mice were sex-matched and randomly assigned to each experimental group. The sample size was determined by Power and Precision (Biostat).

### Purification and aggregation of recombinant human tau RD.

Purification of recombinant human K18 was performed as previously described (48). In brief, His-tagged K18 sequence in the pRK172 expression vector was transformed into *E*. *coli* BL21 (DE3) cells (ABclonal, CD011). The cells were harvested and resuspended in cold buffer A (20 mM Tris, 500 mM NaCl, and 10 mM imidazole, pH 8.0). The mixture was sonicated by an ultrasonic cell disruptor and centrifuged for 20 minutes at 12,000 rpm at 4°C. Then, the supernatants were purified through affinity chromatography with Ni-NTA agarose beads (Cytiva, 17371201). The beads were washed with buffer B (20 mM Tris-HCl, 20 mM imidazole, 0.15 M NaCl, pH 8.0) and eluted by buffer C (20 mM Tris-HCl, 250 mM imidazole, 0.15 M NaCl, pH 8.0). The purity of the protein was confirmed through Coomassie Brilliant Blue staining (Biofroxx, 1912GR025). The endotoxin of the purification of recombinant human K18 was removed using the EtEraser HP kit (Bioendo, ER0015). The purified K18 monomers were lyophilized and resuspended in PBS with 12.5 μM low–molecular weight heparin and 2 mM DTT at a final concentration of 1 mg/mL and then fibrilized by agitation at 1,000 rpm at 37°C for 12 hours. To study the effect of PM2.5 on tau aggregation, PM2.5-tau PFFs were prepared by adding 0.01, 0.05, or 0.1 mg/mL PM2.5 at a final concentration into the tau fibrilization reaction. ThS assay was conducted to evaluate tau aggregation. In brief, PFFs were incubated with 20 μM ThS and read at excitation and emission wavelengths of 440 nm and 480 nm using a SpectraMax microplate reader (Molecular Devices). The monomers and PFFs were stored at –80°C until use.

### TEM.

The morphologies of PFFs were assessed by TEM. The tau PFFs or PM2.5-tau PFFs (1 mg/mL) were dropped onto the 200 mesh carbon-supported copper grids for 3–5 minutes. We removed excess liquid with filter paper, then negatively stained with 2% phosphotungstic acid for 2 minutes and dried at room temperature. The grid was viewed at 80 kV under a transmission electron microscope (HT7800/HT7700, Hitachi).

The synaptic density was also detected by TEM. Mice were perfused with 2% glutaraldehyde. Then, the left hippocampal tissues of mice were postfixed with 1% OsO_4_ for 2 hours at room temperature. After that, the tissues were dehydrated and embedded using standard procedures. Ultrathin sections (90 nm) were stained with 2% uranyl acetate and 2.6% lead citrate, then viewed at 100 kV. Synapses from the DG region were identified by the presence of synaptic vesicles and postsynaptic densities.

### PK digestion.

The tau PFFs and PM2.5-tau PFFs were incubated with PK at a final concentration of 4 μg/mL for various times at 37°C. The interaction was terminated by the addition of protease inhibitor cocktail (Roche, 4693132001). Then the samples were boiled for 10 minutes in 1× SDS loading buffer. The samples were separated on a 10% SDS-PAGE and stained with Coomassie Brilliant Blue solution. The images were analyzed using ImageJ software (NIH). The quantitative measurements were performed of the levels of all remaining protein fragments with PK digestion relative to total proteins without PK digestion by analyzing the gray value of the whole lane.

### Collection of PM2.5.

Referring to previous studies (49, 50), the sampling site was located near a main road in the central urban area of Wuhan, which is a typical urban outdoor point with intensive human activities and traffic flow. A flow air particle sampler (Wuhan Tianhong TH-150C) was set on the rooftop of a building that was 20 m above the ground. The sampler removed PM10 based on the weight of the particles in the cutter and gathered the PM2.5 on a 90 mm diameter glass fiber filter by working at a constant flow rate of 400 L/min. After filtering with 12 gauze layers and lyophilizing, the PM2.5 was resuspended in PBS at a final concentration of 2 mg/mL and then stored at –80°C until use.

### Transfection of cells with PFFs.

Tau-HEK293 cells that stably express GFP-tagged tau RD in HEK293 cells (ATCC, CRL-1573) were cultured in DMEM. The cells were seeded into 6-well plates with coverslips (Thermo Fisher Scientific, 12-545-80) precoated with polyethyleneimine and cultured to a density of approximately 60%. Then, different PFFs were transduced into the cells. For transduction, PBS or different PFFs (4 μg), 96 μL opti-MEM (Gibco, 31985070), and 4 μL Lipofectamine 2000 (Invitrogen, 11668019) were mixed in a tube. After incubation for 20 minutes, the mixtures were directly added to the culture medium.

### Immunofluorescence staining.

For immunostaining, cells were fixed with 4% paraformaldehyde (PFA) solution with 1% Triton X-100 for 10 minutes at room temperature to get rid of soluble tau. The cell slides were then blocked with 5% BSA for 30 minutes. After incubation with primary antibodies overnight at 4°C, the coverslips were incubated in the dark with fluorescence secondary antibody solution at room temperature for 2 hours followed by incubation with DAPI (0.1 μg/mL) solution for 5 minutes. Between each step, the coverslips were washed 3 times with PBS solution. Finally, the coverslips were sealed onto slides using antifade mounting medium (Invitrogen, P36941).

### Primary neuronal culture and treatment.

The isolated primary neurons are cortical neurons prepared from embryos of pregnant tau P301S mice (Jackson Laboratory, stock number 008169) as previously described (51). To evaluate the effects of PM2.5 on tau aggregation, PM2.5 was added directly into the medium of neurons cultured in vitro for 1 week. Tau PFFs were added to the medium 24 hours later. The neurons were harvested for Western blotting or fixed with 4% PFA for immunofluorescence staining. To investigate the seeding activity of pure tau PFFs and PM2.5-tau PFFs in neurons, PBS or different PFFs were added directly into the medium. The phosphorylation and aggregation of tau were detected by Western blot and immunofluorescence staining.

### Immunohistochemistry staining of brain sections.

The mouse brain tissues were fixed with 4% PFA and embedded in paraffin, cut at a thickness of 4 μm, and attached to glass slides. Then the tissue sections were soaked in xylene 3 times for 15 minutes each and rehydrated with decreasing concentrations of ethanol in water. After soaking in tap water for 5 minutes, the tissue sections were placed in citrate buffer (0.1 M sodium citrate, pH 6.0) at 94°C for 20 minutes. The sections were washed 3 times with PBS for 5 minutes each time after the citrate buffer was cooled to room temperature.

For immunohistochemistry staining, the tissue sections were incubated with quenching buffer (3% hydrogen peroxide solution) for 10 minutes followed by blocking with 5% BSA for 30 minutes. After incubating in the primary antibody solution overnight at 4°C, the tissue sections were rinsed in PBS 3 times and incubated with HRP-conjugated secondary antibody solution at 37°C for 1 hour. Next, the tissue sections were incubated with DAB solution, then stained in hematoxylin solution. After dehydrating in 75%, 95%, and 100% ethanol for 5 minutes each and immersing in xylene for 10 minutes, the slides were mounted with a cover glass by neutral balsam.

### Sequential protein extraction.

The cells were lysed on ice in TX-100 buffer before centrifugation at 100,000*g* for 1 hour at 4°C. Then the supernatants containing the TX-soluble fraction were collected and quantified using BCA assay (Invitrogen). The pellets were washed and sonicated twice with TX-100 buffer followed by centrifugation at 100,000*g* for 30 minutes at 4°C each time. The final pellets were resuspended and sonicated with SDS buffer (2% SDS in TBS with protease and phosphatase inhibitors). All samples were normalized by the BCA protein quantitation kit, analyzed by SDS-PAGE, and detected by Western blot analysis.

### Sedimentation analysis.

The PBS solution containing 0.05% Triton X-100 with protease and phosphatase inhibitors was used to lyse the cells. The cell lysate was sequentially centrifuged at 500*g* and 1,000*g* for 5 minutes. Ten percent of the supernatant was set as the total fraction. Then 90% of the supernatant was centrifuged at 100,000*g* at 4°C for 1 hour. The supernatant was collected as the supernatant fraction, and the pellet was washed and sonicated twice with PBS followed by centrifugation at 100,000*g* at 4°C for 30 minutes. Then the pellet was resuspended and sonicated in RIPA buffer containing 2% SDS with protease and phosphatase inhibitors, which was the pellet fraction. All the samples were normalized by BCA protein quantitation and analyzed by Western blot.

### Intranasal instillation of PM2.5.

According to the bulletin from the Wuhan Ecological Environment Bureau, the maximum concentration of PM2.5 in Wuhan in 2021 was 215 μg/m^3^. It has been reported that the conversion factor is 12.3 for converting mouse dose (mg/kg) to human equivalent dose (mg/kg), based on body surface area (52). An individual with 60 kg will breathe in approximately 20 m^3^ of air containing 4,300 μg of PM2.5, resulting in a dose of 71.667 μg/kg/d in humans and an equivalent dose of 881.504 μg/kg/d in mice based on body surface area. Thus, 36 μg PM2.5 in 18 μL PBS was administered to mice by intranasal instillation every 2 days for 4 months, 9 μL in each nostril. To avoid the solution being ingested, mice were placed in the supine position, their heads were manually held at an angle of 70°–90°, and the solution was dripped several times so that PM2.5 would remain in the nasal cavity rather than descending into the pharynx.

### Stereotaxic injection.

Stereotaxic injection of the mouse hippocampus was performed as previously described (53). There are no experimental data to support any lateralization effects in this model. Five micrograms of PFFs (5 μL, 1 mg/mL) were injected into the left hippocampus (anteroposterior –2.5 mm, mediolateral +2.0 mm, dorsoventral –1.7 mm) of 2-month-old tau P301S mice at a rate of 0.1 μL/min using a 10 μL Hamilton syringe. The control mice were injected with 5 μL of sterile PBS. After injection, the syringe was maintained for 5 minutes for complete absorption of the PBS or PFFs. Postoperative care was provided until the mice recovered from anesthesia.

### Morris water maze.

Three months after stereotaxic injection, 5-month-old mice were tested by Morris water maze, which is a reliable tool for assessing spatial learning and memory but not sensitive to spatial working memory assessment (54). During the training phase, mice were trained with extra maze cues. Each subject was tested 4 times per day to reach the platform within 60 seconds. If the mouse failed to find the platform within 60 seconds, it was manually guided to the platform and allowed to remain there for 15 seconds. On the first day, visual training (a visible flag was placed on the platform only on the first day) was performed. Then, each mouse was tested for 5 consecutive days without a visible flag. The probe trial was performed after the training test, in which the platform was removed and the escape latency and the time spent in the platform quadrant were recorded and analyzed by ANY-Maze software.

### Y-maze test.

The Y-maze test can better assess changes in spatial working memory, which is mainly regulated by the hippocampus and the prefrontal cortex (55). Y-maze is easily disturbed by the environment, but it makes up for the deficiency of the Morris water maze test. Each arm was 40 cm long, 12 cm high, 3 cm wide at the bottom, and 10 cm wide at the top. The arms converged in an equilateral triangular central area that was 4 cm at its longest axis. The 3 arms were randomly designated. Start arm: The mouse started to explore when the arms were always open. Novel arm: The arm was blocked during the first trial but opened during the second trial. The test consisted of 2 trials separated by an intertrial interval (ITI) to assess spatial recognition memory. The first trial (training) lasted for 5 minutes, and the mice were allowed to explore only 2 arms (start arm and another arm) in the maze, with the third arm (novel arm) being blocked. The series of arm entries was recorded visually. After a 1-hour ITI, the second trial (retention) was conducted, during which all 3 arms were accessible, and novelty versus familiarity was analyzed. In the second trial, the mice were placed back in the maze in the same starting arm, with free access to all 3 arms for 5 minutes. Recordings were taken and analyzed using the ANY-Maze software. Then the number of entries and time spent in each arm were analyzed.

### Electrophysiology.

Electrophysiology of hippocampal neurons was performed using a whole-cell patch-clamp as described previously (56). Briefly, the murine brains were placed in precooled, oxygenated cutting solution (2.5 mM KCl, 25 mM d-glucose, 25 mM NaHCO_3_, 1.26 mM NaH_2_PO_4_, 0.5 mM CaCl_2_, 7.2 mM MgCl_2_, 3.1 mM Na-pyruvate, 97 mM choline chloride, and 11.35 mM ascorbic acid, pH = 7.3–7.4) and cut along the sagittal direction at 350 μm thickness by using a Leica VT1200S vibratome. Then, the brain slices containing the dorsal hippocampus were incubated with oxygenated artificial cerebrospinal fluid (118 mM NaCl, 2.5 mM KCl, 26 mM NaHCO_3_, 1 mM NaH_2_PO_4_, 2 mM CaCl_2_, 2 mM MgCl_2_ and 22 mM glucose, pH = 7.3–7.4) for 1 hour at room temperature. The recording microelectrodes were made with borosilicate glass (Warner Instruments, Inc.) by using a horizontal drawing puller (Sutter Instrument) to form a high-resistance seal (GΩ) between the recording microelectrodes and the cell membrane, containing an intracellular solution with a resistance of 6 MΩ (20 mM KCl, 100 mM CsCH_3_SO_3_, 4 mM Mg-ATP, 0.3 mM Tris-GTP, 10 mM HEPES, 7 mM Tris_2_-Phosphocreatine, 3 mM QX-314, pH = 7.3–7.4, osmolarity = 298 mOsm). The fEPSPs in CA1 neurons were recorded by stimulating the Schaeffer fibers from CA3. Three trains of high-frequency stimulation (100 Hz, 1-second duration) were applied to induce LTP. Electrical signals were recorded by an Axon Instruments clamp 700B amplifier at 25 kHz and digitalized with a Digidata 1,440 digitizer (Molecular Devices) controlled by Clampex 10.1 (Molecular Devices).

### Golgi staining.

Golgi staining of the hippocampus was performed using a Golgi staining kit (FD Neuro Technologies, Inc., PK-401). In brief, the brain tissue was soaked in the silver impregnation solution for 2 weeks in the dark and then incubated with stock solution for 3–7 days. The samples were cut at 60 μm and attached on gelatin-coated glass slides followed by incubation with staining solution for 10 minutes. After rinsing in double-distilled water, the sections were dehydrated through 95% and 100% ethanol, cleared in xylene, and mounted with mounting medium. Images of dendritic spines were taken at 100× original magnification using an Axioplan (ZEISS) microscope. The spine density was counted in all clearly evaluable areas of 50~100 μm of secondary dendrites from each imaged DG neuron.

### Statistics.

Data were expressed as means ± SEM and analyzed with GraphPad Prism (version 8.0). Two-group comparisons were performed with 2-tailed Student’s *t* tests. Comparisons among 3 groups or more than 3 groups were performed with 1-way ANOVA followed by Tukey’s multiple comparisons for post hoc tests. For the time course studies, the level of significance was determined using the 2-way ANOVA and Holm-Šídák multiple comparisons test. Differences with *P* values less than 0.05 were considered significant.

### Study approval.

All experiments involving animal use were approved by the Laboratory Animal Welfare Ethical Committee of Renmin Hospital of Wuhan University (No. 20210402).

### Data availability.

Supporting data values for each figure are made available in the Supporting Data Values file.

## Author contributions

Congcong Liu and LM contributed equally to this work as co–first authors. Congcong Liu is listed first of the co–first authors for performing the majority of the manuscript drafting, editing, revising, and formatting following acceptance. ZZ conceived the project and designed the experiments. Congcong Liu and LM performed most of the experiments. JC performed the electrophysiology experiments. YG, MZ, MX, TX, and XG assisted with molecular biology experiments and animal experiments. LM, Chaoyang Liu, and TL performed the data analysis.

## Supplementary Material

Supplemental data

Unedited blot and gel images

Supporting data values

## Version 1 07/22/2024

Electronic publication
