# Supplementary material for: PM2.5 triggers tau aggregation in a mouse model of tauopathy
Source: JCI Insight. 2024 Jul 22;9(14):e176703. doi: 10.1172/jci.insight.176703 (PMC11383351; doi:10.1172/jci.insight.176703)
Supplement: Supplemental data [file jciinsight-9-176703-s007.pdf]

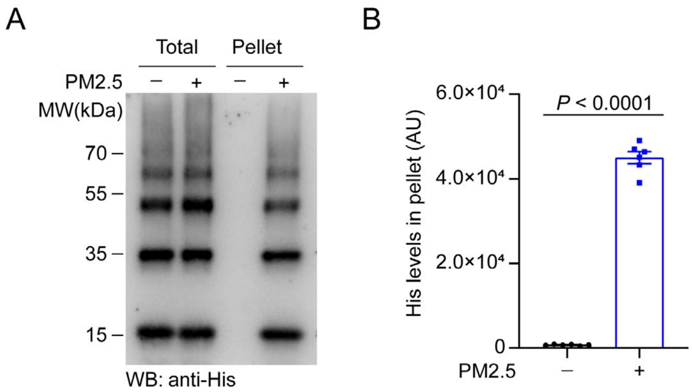

Supplementary Figure 1 Fine particulate matter (PM2.5) interacts with Tau.

(A, B) Sedimentation analysis showing the interaction between PM2.5 and Tau. **(A)** Western blots assay of His. **(B)** The bar graph shows the quantification of the His levels in the pellet. Data are presented as mean ± SEM. *P*-values were determined by Student's *t*-test. n = 6 biologically independent experiments. AU, arbitrary units.

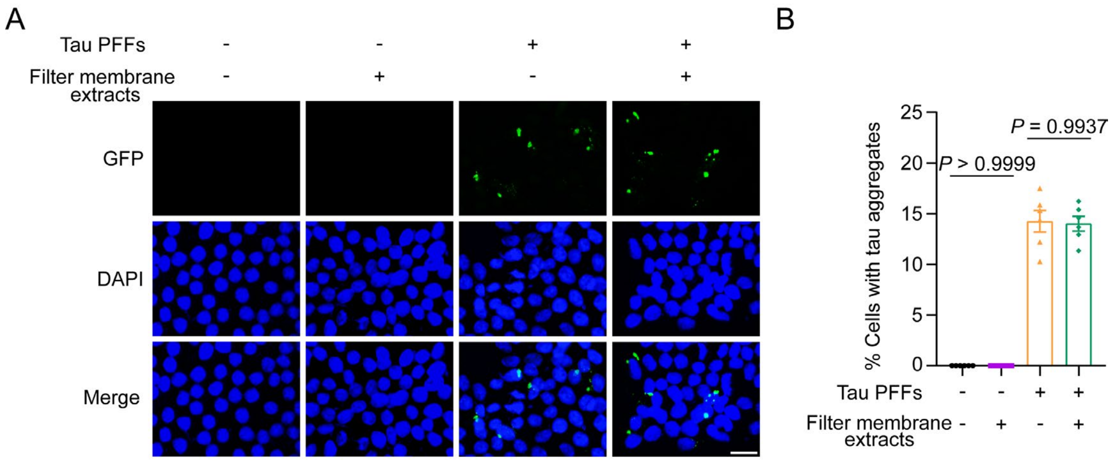

Supplementary Figure 2 Filter membrane extracts without collection of PM2.5 have no effect on tau aggregation.

(A, B) Tau-HEK293 cells were pre-treated with or without the filter membrane extracts for 24 h, and transduced with PBS or Tau preformed fibrils (PFFs). **(A)** Shown are insoluble Tau aggregates at 48 h post-transduction. Scale bar: 20 µm. **(B)** Quantification of insoluble Tau aggregates in Tau-HEK293 cells. n = 6 independent biological experiments (each point represents the average of 10 random fields from each experiment). Data are presented as mean ± SEM. *P*-values were determined by one-way ANOVA followed by Turkey's multiple comparisons test.

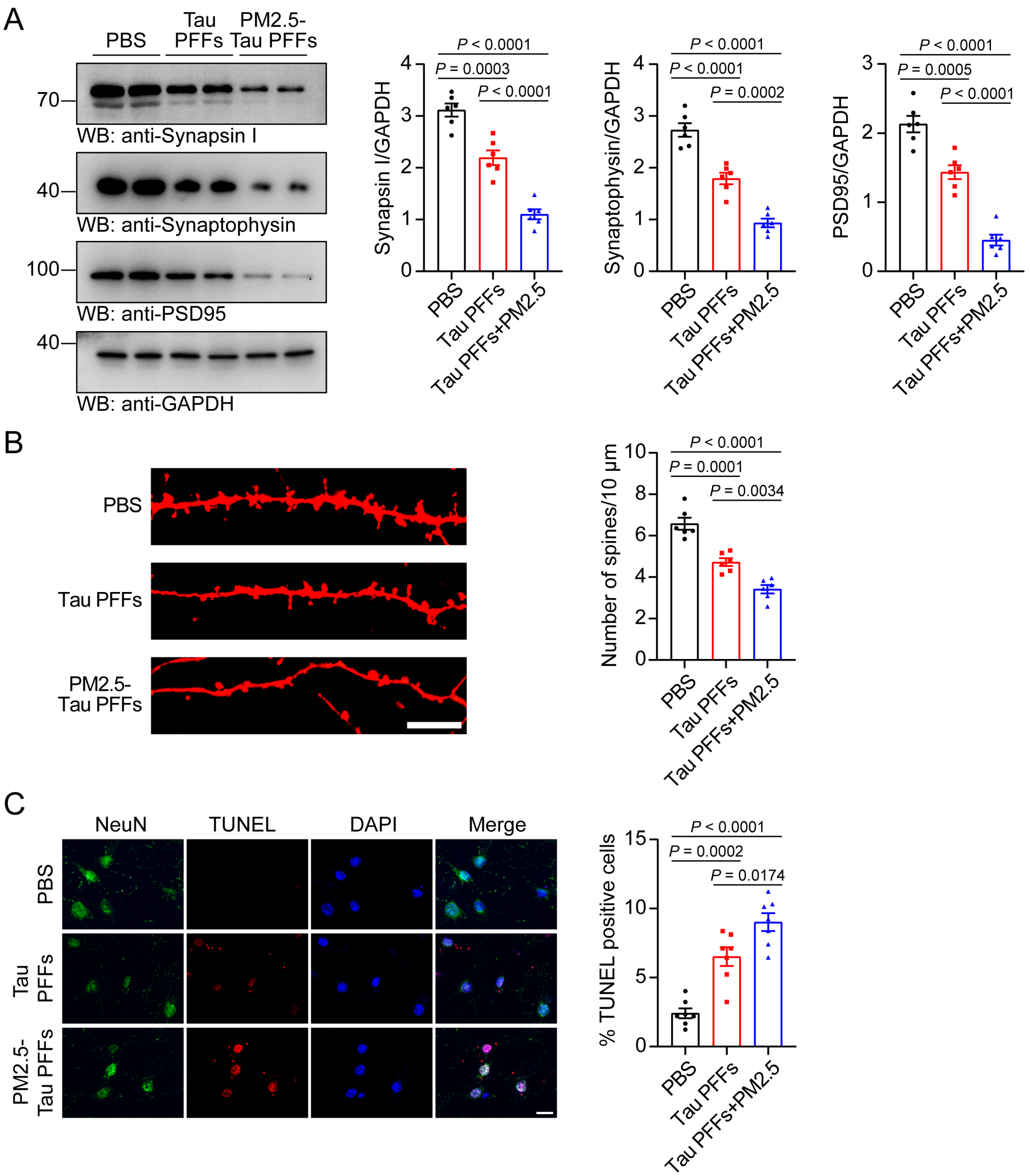

Supplementary Figure 3 PM2.5-Tau PFFs induce synaptic degeneration.

(A) The levels of synapsin I, synaptophysin, and PSD95 in neurons exposed to PBS, Tau PFFs, or PM2.5-Tau PFFs for 10 days. Right: The bar graph shows the quantification of synapsin I, synaptophysin, and PSD95 expression levels. n = 6 independent experiments.

(B) DiI staining of neurons exposed to PBS, pure Tau PFFs, or PM2.5-Tau PFFs for 10 days. Right: The bar graph shows the quantification of spine density. n = 6 independent

biological experiments (each point represents the average of 10 random fields from each experiment). Scale bars: 10 μm.

(C) Double immunofluorescence of NeuN and TUNEL in neurons treated with PBS, pure Tau PFFs, or PM2.5-Tau PFFs for 10 days. Right: The bar graph shows the quantification of the percentage of apoptotic cells. n = 6 independent biological experiments (each point represents the average of 10 random fields from each experiment). Scale bars: 20 μm.

Data are presented as mean ± SEM. *P*-values were determined by one-way ANOVA followed by Turkey's multiple comparisons test.

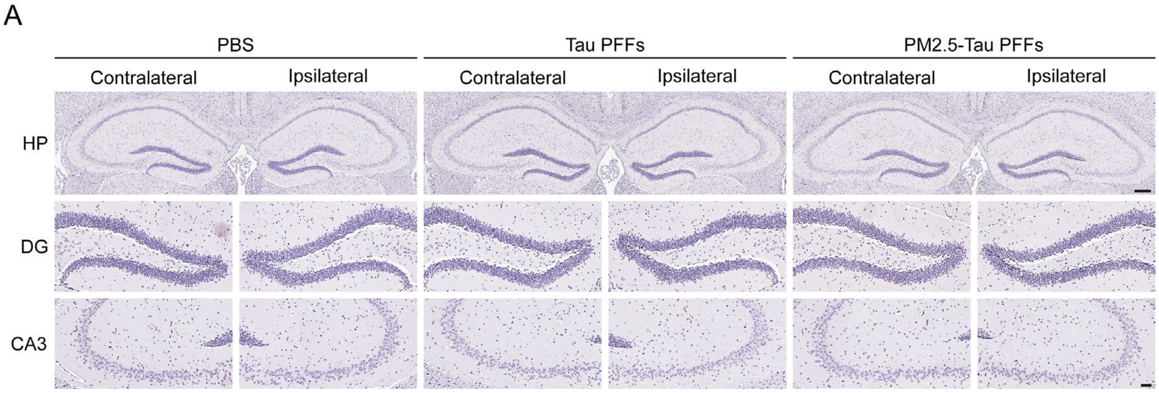

Supplementary Figure 4 PM2.5-Tau PFFs have no effect on the propagation of Tau pathology in WT mice.

(A) Immunohistochemistry of phosphorylated Tau (AT8) in WT mice at 3 months after the injection of PBS, Tau PFFs, or PM2.5-Tau PFFs. Representative images of p-Tau (AT8) staining in the dentate gyrus and CA3 of the mouse hippocampus. Scale bars, 200 μm in the top panel, and 50 μm in the lower panels.

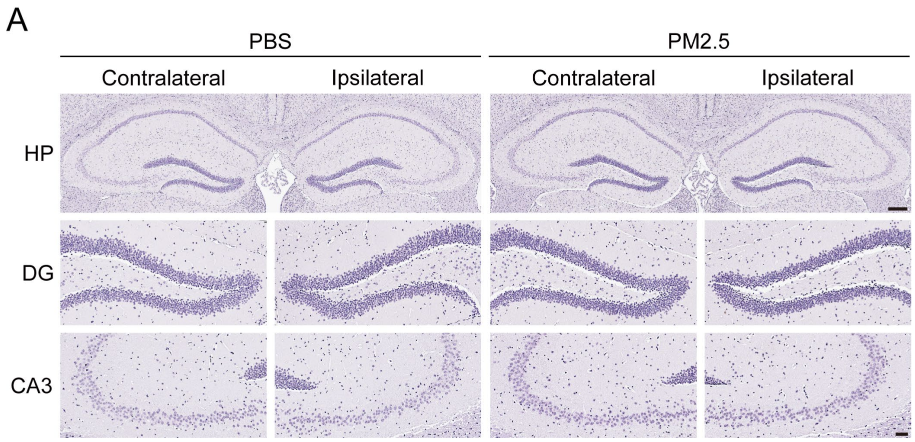

Supplementary Figure 5 Intranasal instillation of PM2.5 have no effect on Tau pathology in WT mice.

(A) Immunohistochemistry of phosphorylated Tau (AT8) in WT mice treated with PM2.5 for 4 months. Representative images of p-Tau (AT8) staining in the dentate gyrus and CA3 of the mouse hippocampus. Scale bars, 200 μm in the top panel, 50 μm in the lower panels.
