# Supplementary material for: PM2.5 triggers tau aggregation in a mouse model of tauopathy
Source: JCI Insight. 2024 Jul 22;9(14):e176703. doi: 10.1172/jci.insight.176703 (PMC11383351; doi:10.1172/jci.insight.176703)
Supplement: Unedited blot and gel images [file jciinsight-9-176703-s008.pdf]

Full unedited gel for Figure 1D

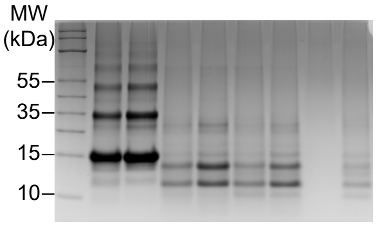

Full unedited blots for Figure 2D

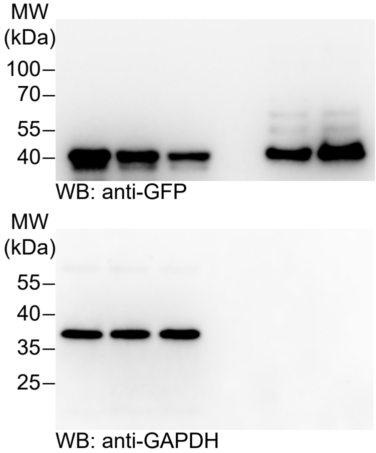

Full unedited blots for Figure 2F

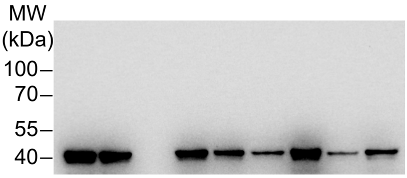

Full unedited blots for Figure 2L

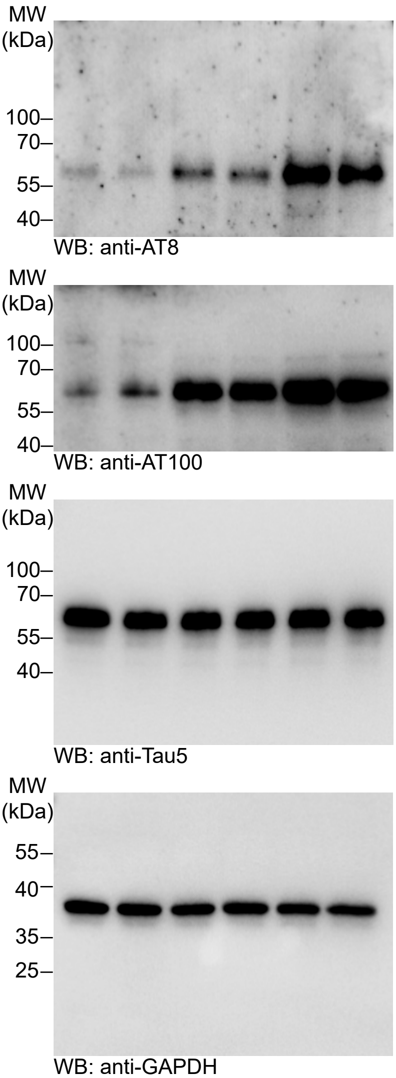

Full unedited blots for Figure 3C

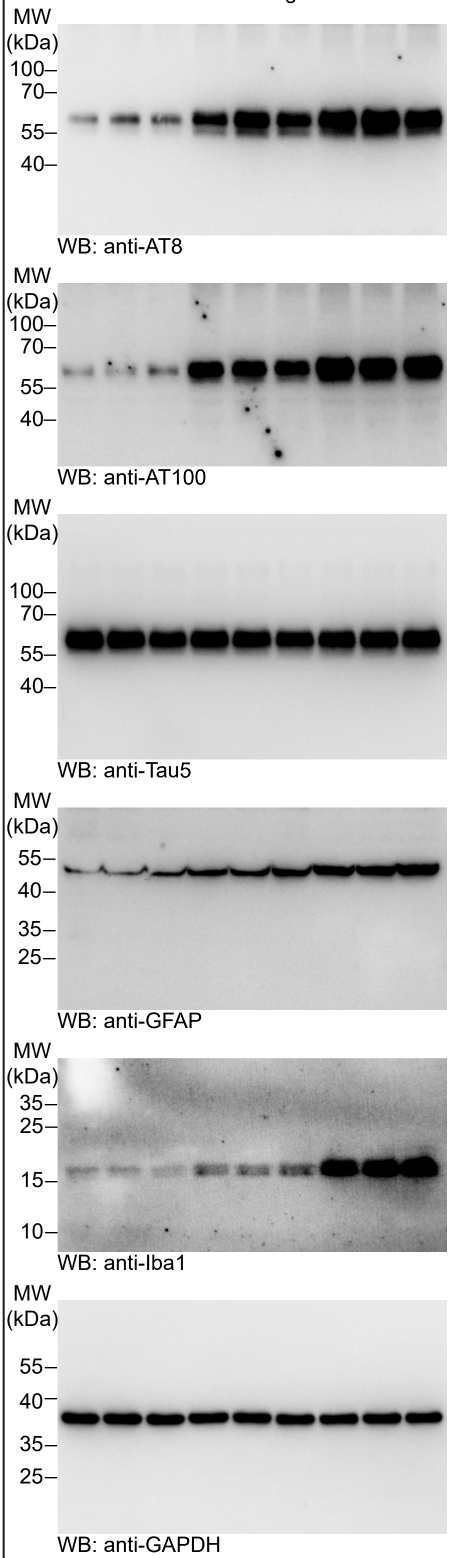

Full unedited blots for Figure 5E

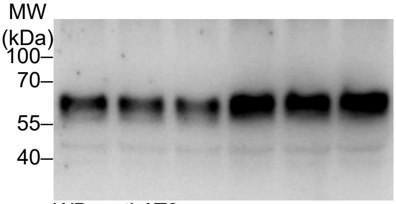

WB: anti-AT8

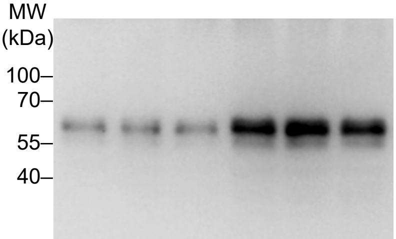

WB: anti-AT100

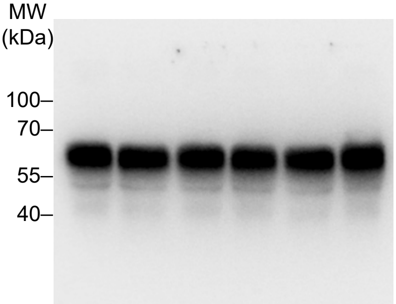

WB: anti-Tau5

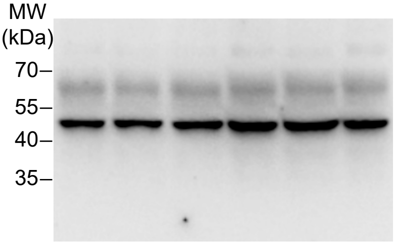

WB: anti-GFAP

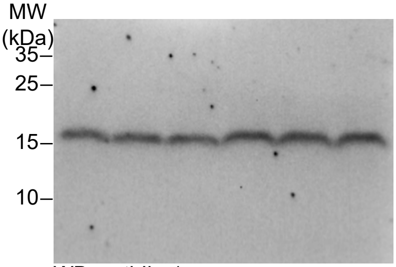

WB: anti-Iba1

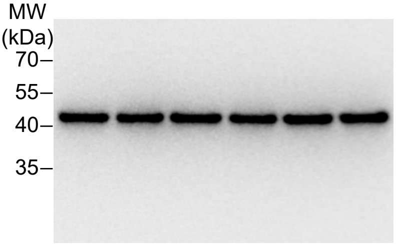

WB: anti-β-actin

Full unedited blots for Figure S1A

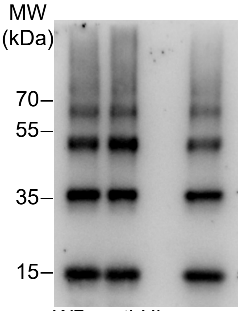

WB: anti-His

Full unedited blots for Figure S3A

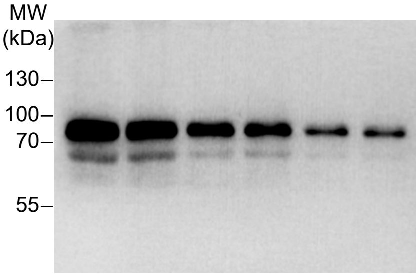

WB: anti-Synapsin I

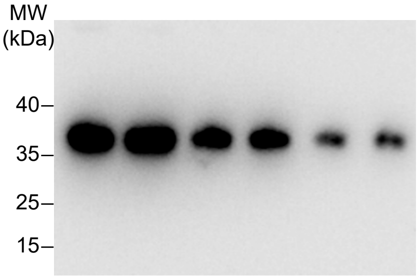

WB: anti-Synaptophysin

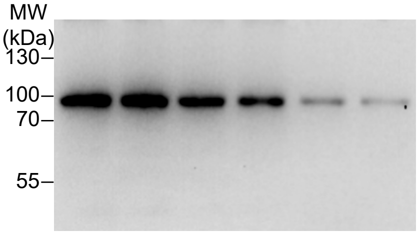

WB: anti-PSD95

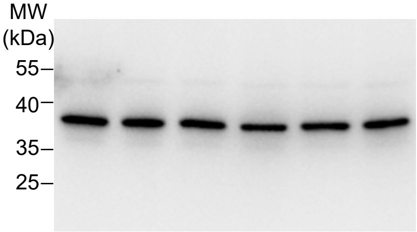

WB: anti-GAPDH
